# Cost-effectiveness of serological tests for human visceral leishmaniasis in the Brazilian scenario

**DOI:** 10.1371/journal.pntd.0008741

**Published:** 2020-10-08

**Authors:** Mariana Lourenço Freire, Aline de Souza, Gláucia Cota, Ana Rabello, Tália Machado de Assis

**Affiliations:** 1 Pesquisa Clínica e Políticas Públicas em Doenças Infecciosas e Parasitárias, Instituto René Rachou, Fundação Oswaldo Cruz, Barro Preto, Belo Horizonte, Minas Gerais, Brasil; 2 Centro Federal de Educação Tecnológica de Minas Gerais, Campus Contagem, Alameda das Perdizes, Cabral, Contagem, Minas Gerais, Brasil; Federal University of Ceará, Fortaleza, Brazil, BRAZIL

## Abstract

Human visceral leishmaniasis (VL) is a severe and potentially fatal parasitic disease if not correctly diagnosed and treated. Brazil is one of the three countries most endemic for VL and, like most countries affected by this disease, has a large budget constraint for the incorporation of new health technologies. Although different diagnostic tests for VL are currently available in the country, economic studies evaluating diagnostic kits are scarce. The objective of this study was to conduct a cost-effectiveness analysis of the nine available diagnostic tests for human VL in HIV-infected and uninfected patients in Brazil. The perspective of analysis was the Brazilian public health system, and the outcome of interest was "cases diagnosed correctly". The costs of the tests were estimated using the microcosting technique, and comparisons were performed with decision trees. Sensitivity analyses were explored applying variations in cost and effectiveness values. For VL diagnosis among HIV-uninfected patients, using blood samples for the rapid tests (RDTs), the noncommercial direct agglutination test (DAT-LPC) and IT-LEISH were cost-effective tests compared with the baseline OnSite test, but they presented different incremental cost-effectiveness ratios (ICER) of US$7.04 and US$ 205.40, respectively. Among HIV-infected patients, DAT-LPC was the most cost-effective diagnostic test. Comparisons among the tests with the same methodology, based on the low ICER values, revealed that IT-LEISH was the most cost-effective test among the RDTs and the Ridascreen Leishmania Ab among the ELISA tests. These results confirm that cost-effectiveness analyses can provide useful information to support the incorporation of new health technologies within a known scenario and willingness to pay threshold. It was observed that tests based on the same methodologies presented different cost-effectiveness ratios for the same group of patients and that different tests should be recommended for different patient groups. DAT-LPC was an important cost-effective strategy for all patients, requiring minimum laboratorial infrastructure, and IT-LEISH was the cost-effective test for VL screening in HIV-uninfected patients. IT-LEISH and DAT-LPC have complementary profiles and should both be provided by the Brazilian health system.

## Author summary

Human visceral leishmaniasis (VL) is a neglected illness affecting economically vulnerable populations in more than 80 countries. Approximately 90% of the cases registered in Latin American occur in Brazil, where more than 7% of cases are also HIV-infected, and the public health system sustains the costs of illness. Considering the importance of correct diagnosis for VL control and for the optimal use of limited public health resources, we present a cost-effectiveness analysis of the available tests in Brazil up to 2019, stratified according to HIV status. In the different analyses performed, a national noncommercial direct agglutination test (DAT-LPC) and the rapid test IT LEISH were the most cost-effective diagnostic tests. DAT-LPC is especially cost-effective for VL screening among HIV-infected patients, presenting the lowest cost and highest effectiveness. DAT-LPC and IT LEISH are tests that can be performed in laboratories with minimal installed infrastructure, allowing cost-effective strategies for the diagnostic investigation of cases with VL symptoms. The results confirm the importance of cost-effectiveness analyses prior to the incorporation of new health technologies.

## Introduction

Visceral leishmaniasis (VL) is an important public health problem that mainly affects low-income populations in countries with precarious social and economic development. According to the World Health Organization (WHO), roughly 20,000 new cases of this disease are reported annually, and 73% are located in India, Brazil, Sudan and South Sudan [1]. Brazil accounts for approximately 96% of VL cases recorded in Latin America, with an average of 4,120 cases and 274 deaths per year reported between 2007 and 2017 [2,3]. The occurrence of VL in individuals infected with human immunodeficiency virus (HIV) corresponds to 7.4% of cases of VL in Brazil and represents an additional challenge [3,4]. Low-sensitivity serological tests, lower cure rates, and higher relapse and mortality rates are VL trademarks among HIV-infected individuals [4–6].

The main objective of the Brazilian Surveillance and Control Program for Visceral Leishmaniasis is the reduction of the case–fatality rate through early diagnosis and appropriate treatment. Diagnosis of VL is performed by the combination of epidemiological information and clinical findings in addition to parasitological or serological tests, especially rapid diagnostic tests (RDTs). Currently, parasitological examinations of bone marrow aspirate, immunofluorescence antibody test (IFAT—IFI Leishmaniose Humana Fiocruz) and RDTs are performed and/or made available by public health services in Brazil [7]. Polymerase chain reaction (PCR) is restricted to reference centers, and immunoenzymatic assays (ELISAs) are mainly used in private health laboratories. IFAT was the most widely used serological diagnostic test in Brazil until 2013, when 46% of VL cases were confirmed using this test. RDTs were introduced in Brazil from 2009. After 2014, although IFAT was used in the same 46% of confirmed VL cases, according to the Brazilian diseases reporting system (SINAN), RDT was performed in more than 50% of VL cases [7]. In recent years, a progressive reduction in the use of parasitological examination has been observed in Brazil, although the percentage of this invasive test of approximately 34% of VL cases in 2017 reveals that the strategy of rapid tests is not reaching its target.

An ideal diagnostic strategy requires a balance between safety, technique performance, and cost. In a country such as Brazil, where health care is considered as a universal right to be provided by the public health system, the economic discussions are especially important [8]. In this sense, cost-effectiveness analyses are important tools to support public health decision making for an appropriate allocation of health resources [9], describing interventions in terms of cost per unit of health gain [10,11].

Recently, Freire et al. (2019) identified six registered and commercially available serological tests for the diagnosis of VL in Brazil and evaluated their performance in different groups of patients [12]. As a step forward in the direction of evidence-based decision making, the objective of the present study was to perform a cost-effectiveness analysis of available tests in the country for the diagnosis of VL according to the patient’s HIV status.

## Methodology

### Study design

The cost-effectiveness analysis for nine diagnostic tests for VL was performed using an analytical decision model based on decision trees developed in TreeAge Pro 2015 software (TreeAge Software, Inc., Massachusetts, United States).

The effectiveness was defined as a VL case correctly diagnosed, and the analytic horizon of this analysis started at the first clinical evaluation of a patient with suspected VL and ended at the time of diagnosis confirmation. The definition of a VL-suspected case was a patient with fever associated with at least one of the following signs: splenomegaly, hepatomegaly, anemia, leukopenia or thrombocytopenia.

### Diagnostic interventions

Six registered and commercially available tests for VL diagnosis of the Brazilian agency for registration of health products (ANVISA) in January 2017 were included in the analyses (Table 1). Additionally, the OnSite *Leishmania* IgG/IgM Combo, Kalazar Detect and a direct agglutination test improved in Brazil (DAT-LPC) were included. OnSite *Leishmania* IgG/IgM Combo was included because it was the RDT available for the diagnosis of VL in the public Brazilian Unified Health System (SUS) at the time of analysis. Kalazar Detect was included in the analyses because it was the first RDT available in Brazil. The inclusion of DAT-LPC, despite its being a noncommercial test, is justified due to its high performance (sensitivity ranging from 96.6 to 99% and specificity ranging from 96.3 to 97.5%) [13], as it was considered cost-effective in an economic analysis previously conducted in Brazil [14]. Furthermore, the test has the potential to be produced in the country, reducing the risk of shortage, a nonrare problem related to the importation process.

**Table 1:** Diagnostic kits for human visceral leishmaniasis included in the study.

Diagnostic tests	Manufacturer	Country	Record Status	Methodology
IT LEISH	BIO-RAD Laboratories, Inc	France	Effective	RDT
OnSite *Leishmania* IgG/IgM Combo	CTK Biotech Inc.	China	Effective	RDT
IIF Leishmaniose Humana	Fiocruz	Brazil	Effective	IFAT
*Leishmania* IFA IgG	Vircell S.L.	Spain	Effective	IFAT
*Leishmania* ELISA IgG+IgM	Vircell S.L.	Spain	Effective	ELISA
Ridascreen *Leishmania* Ab	R-Biopharm AG	Germany	Effective	ELISA
NovaLisa *Leishmania infantum* IgG	Novatec Immundiagnostica GMBH	Germany	Effective	ELISA
Kalazar Detect Rapid Test	Inbios International, Inc.	United States	Expired	RDT
DAT-LPC	Fiocruz Minas	Brazil	No record	Direct agglutination test

RDT–rapid diagnostic test; IFAT–Immunofluorescence reactions; ELISA–Immunoenzymatic assays.

### Effectiveness

The test effectiveness is calculated by the number of correct results obtained with a test, meaning the sum of the true positive and true negative results. In turn, the performance of the serological VL diagnostic tests refers to their sensitivity (true positives and false negatives) and specificity (true negatives and false positives) and was previously determined by Freire et al. (2018 and 2019) [12,15] using well-characterized panels of samples from Brazilian patients with clinical suspicion of VL, infected and uninfected by HIV (Table 2). In these panels, all the VL-cases were parasitologically confirmed (bone marrow smear or culture), and the controls were represented by symptomatic patients with negative parasitological tests for *Leishmania* and confirmation of other disease. For one test, the OnSite *Leishmania* IgG/IgM Combo, there is no performance data in HIV-infected patients; thus, it was not included in this subgroup cost effectiveness analysis.

**Table 2:** Performance of the diagnostic tests stratified according to HIV status based on a previous study.

Diagnostic tests	HIV-UNINFECTED PATIENTS Sensitivity (%) (CI 95%) Specificity (%) (CI 95%)	HIV-INFECTED PATIENTS Sensitivity (%) (CI 95%) Specificity (%) (CI 95%)
**IT LEISH**	96.3 (89.6–98.7)	63.2 (47.3–76.6)
	96.2 (89.4–98.7)	97.4 (86.8–99.6)
**OnSite *Leishmania* IgG/IgM Combo**	91.2 (84.5–95.1)	-
	94.5 (86.7–97.9)	-
**IIF Human Leishmaniasis**	86.3 (77.0–92.2)	60.5 (44.7–74.4)
	82.3 (72.4–89.1)	89.7 (76.4–95.9)
***Leishmania* IFA IgG**	78.8 (68.6–86.3)	60.5 (44.7–74.4)
	96.2 (89.4–98.7)	92.3 (79.7–97.4)
***Leishmania* ELISA IgG+IgM**	77.5 (67.2–85.3)	63.2 (47.3–76.6)
	93.7 (86.0–97.3)	97.4 (86.8–99.6)
**Ridascreen Leishmania Ab**	93.8 (86.2–97.3)	78.9 (63.7–88.9)
	77.2 (66.8–85.1)	89.7 (76.4–95.9)
**NovaLisa *Leishmania infantum* IgG**	86.3 (77.0–92.2)	65.8 (49.9–78.8)
	96.2 (89.4–98.7)	94.9 (83.1–98.6)
**Kalazar Detect**	92.5 (84.6–96.5)	47.4 (32.5–62.7)
	94.9 (87.7–98.0)	97.4 (86.8–99.6)
**DAT–LPC**	93.8 (86.2–97.3)	89.5 (75.9–95.8)
	97.5 (91.2–99.3)	89.7 (76.4–95.9)

Source: Freire et al. (2018 and 2019) [12,15].

### Direct costs

Direct costs were estimated by microcosting, a technique that consists of a detailed enumeration of direct costs from every resource consumed separately for each diagnostic test execution [16,17]. Only operational costs were estimated, assuming that all investments in infrastructure and equipment were unnecessary. The following items were included in costs estimation:

- Unit value of the test: for IT LEISH and OnSite Leishmania IgG/IgM Combo, data were obtained from the Electronic Reverse Auction n° 00066/2017-000; unit costs of DAT-LPC have been previously reported [14], and for the other tests, data were obtained from test distributor companies in Brazil, considering all import costs;
- Laboratory technician salaries: costs were calculated according to the time spent collecting the biological material and performing the test. As the reference for this cost, the salaries of health professionals of the municipality of Belo Horizonte, Minas Gerais, Brazil were used [18];
- Individual protection equipment (gloves and masks) and materials for blood collection (alcohol solution, cotton, needle, syringe, and blood collection tubes): these costs were obtained from the Brazilian Health System Reimbursement Values Table [19];
- Consumables (sterilized filter tips and microscope lamps) and equipment maintenance (centrifuge, heating incubator, refrigerator, fluorescence microscope and pipettes calibration): these costs were obtained from the business contract sector of one Brazilian health institute (Oswaldo Cruz Foundation—Fiocruz).

All estimates were performed based on costs in January 2018 and were originally estimated in Brazilian currency (Real—R$), subsequently converted into US dollars (US$ = 4.31 on 11 February 2020) [20]. The cost details of the diagnostic tests included in the analyses are presented in the Supplementary material (S1 Table).

### Cost-effectiveness analytical model

**Fig 1:**
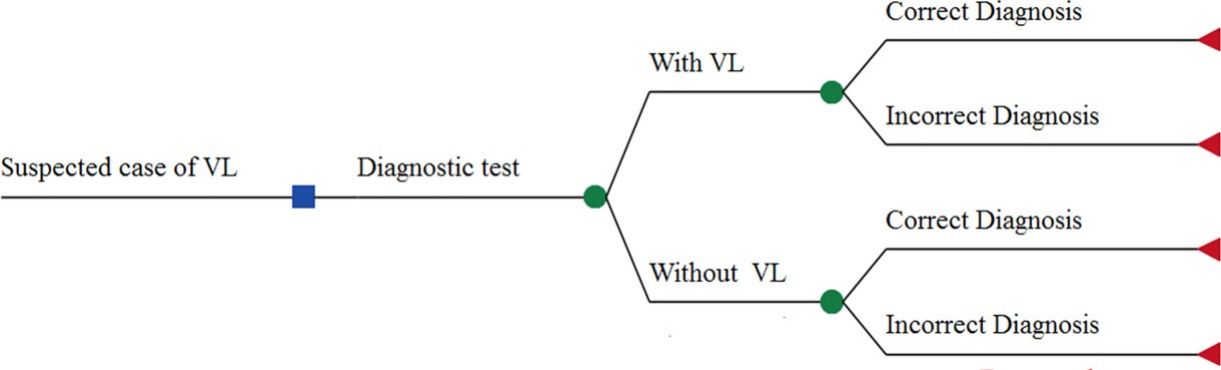
Basic structure of the decision tree used to compare diagnostic tests in suspected cases of visceral leishmaniasis in a reference center for the disease.

The perspective of the analysis was the Brazilian public health system, and the outcome of interest was "cases diagnosed correctly". The prevalence of the disease, meaning the probability of a suspected case being a true VL-case, was estimated at 67% based on a previous study in Brazil [21]. In the decision trees, four outcomes were possible: VL correctly diagnosed (true positive), VL incorrectly diagnosed (false negative), individual without VL correctly diagnosed (true negative), and individual without VL incorrectly diagnosed (false positive). The basic structure of the decision tree is shown in Fig 1.

Initially, cost-effectiveness analyses were performed for all nine diagnostic tests together, and further analysis was performed according the methodology of the tests: ELISAs, RDTs and IFATs. In all analyses, including IT LEISH and OnSite Leishmania IgG/IgM Combo, the cost of tests according to the type of biological sample (blood and serum) were included, and the effectiveness in these different samples was considered similar [22]. All analyses were stratified according to the condition "HIV infection", present or absent.

For the cost-effectiveness analysis, the diagnostic tests were sorted from the least to the most expensive test; thus, the test presented in the first line or baseline is always the less costly test and the initial comparator in the analysis. A new technology presenting lower effectiveness and increased cost is referred to as absolutely dominated (AbD). In the common scenario, wherein an improvement on the effectiveness of the technology is associated with an increased cost, an undominated (ND) technology is referred [23]. The incremental cost-effectiveness ratio (ICER) is calculated using only the strategies on the cost-effectiveness frontier and is calculate by dividing the incremental cost of the diagnostic strategy by its incremental effectiveness. These results are expressed in cost per case correctly diagnosed and represent the additional expense due to a correct diagnosis. The incremental values were always calculated for each test using as comparator the previous less costly and most effective test. In other words, the comparator is variable during analyses. A new technology presenting higher cost and a lower ICER value than the last dominant strategy is referred to as a weak dominant (WD) strategy [24].

### Sensitivity analysis

To test how much the conclusions would resist variations of the parameters included in the analyses, univariate sensitivity analyses were conducted, varying the main parameters used in the model: sensitivity, specificity and costs. The sensitivity and specificity rates of the tests were varied considering the 95% confidence interval of the parameter, according to that previously estimated [12,15] (Table 2). The costs were varied arbitrarily by ± 25%.

## Results

The cost-effectiveness analyses for HIV-uninfected patients are shown in 5 3. Comparing all tests, and considering OnSite Leishmania IgG/IgM Combo and IT LEISH performed in blood samples (Table 3), DAT-LPC presented an ICER of US$ 7.04 per case correctly diagnosed compared to OnSite Leishmania IgG/IgM Combo, the least expensive test. In turn, IT LEISH exhibited an incremental effectiveness in relation to DAT-LPC at a much higher cost, with an ICER of US$ 205.40 per case correctly diagnosed. All the other tests were more expensive and less effective. Comparing all the tests performed in serum samples (Table 3), the ICER of IT LEISH compared to that of DAT-LPC was still higher, at US$ 399.55 per case correctly diagnosed. The sensitivity analyses for these comparisons are presented in the Supplementary material (S2 Table).

**Table 3:** Cost-effectiveness analysis of diagnostic tests for visceral leishmaniasis for HIV-uninfected patients.

**Cost of OnSite Leishmania IgG/IgM Combo and IT LEISH performed in blood**						
**Diagnostic tests**	**C(US$)**	**IC (US$)**	**E**	**IE**	**ICER (US$)**	**DM**
OnSite Leishmania IgG/IgM Combo^α^	3.48		0.92			
DAT-LPC	3.72	0.21	0.95	0.03	7.04	ND
Kalazar Detect	5.01	1.29	0.94	-0.02	*	AbD
IT LEISH	5.12	1.40	0.96	0.01	205.40	ND
IFI Leishmaniose Humana	8.06	2.94	0.85	-0.11	*	AbD
Ridascreen *Leishmania* Ab	9.53	4.41	0.88	-0.08	*	AbD
*Leishmania* ELISA IgG+IgM	11.94	6.82	0.83	-0.13	*	AbD
*Leishmania* IFA IgG	12.30	7.18	0.85	-0.11	*	AbD
NovaLisa *Leishmania infantum* IgG	17.04	11.92	0.89	-0.07	*	AbD
**Cost of all tests performed in serum**						
**Diagnostic tests**	**C (US$)**	**IC (US$)**	**E**	**IE**	**ICER (US$)**	**DM**
DAT-LPC^α^	3.72		0.95			
Kalazar Detect	5.01	1.29	0.94	-0.02	*	AbD
OnSite Leishmania IgG/IgM Combo	5.10	1.37	0.92	-0.03	*	AbD
IT LEISH	6.44	2.72	0.96	0.01	399.55	ND
IFI Leishmaniose Humana	8.06	1.62	0.85	-0.11	*	AbD
Ridascreen *Leishmania* Ab	9.53	3.09	0.88	-0.08	*	AbD
*Leishmania* ELISA IgG+IgM	11.94	5.50	0.83	-0.13	*	AbD
*Leishmania* IFA IgG	12.30	5.86	0.85	-0.11	*	AbD
NovaLisa *Leishmania infantum* IgG	17.04	10.60	0.89	-0.07	*	AbD

C: cost (cost value for the diagnostic tests); IC: incremental cost (difference in cost between a diagnostic tests and the previous less costly test on the cost-effectiveness frontier); E: effectiveness (effectiveness value for the diagnostic test); IE: incremental effectiveness (difference in effectiveness between a diagnostic test and the previous less costly test on the cost-effectiveness frontier); ICER: incremental cost-effectiveness ratio (the incremental cost-effectiveness ratio comparing a diagnostic test to the previous less costly test on the cost-effectiveness frontier); DM: dominance; ND: undominated; AbD: absolutely dominated ^α^baseline or first comparator

*negative ICER values.

For HIV-infected patients, the cost-effectiveness analyses are shown in Table 4. Considering all diagnostic tests evaluated and the costs of IT LEISH using blood or serum samples, DAT-LPC was the most cost-effective test, presenting the lowest cost (direct cost: US$ 3.72) and highest effectiveness (90%) (Table 4 - Comparison 1). Variations in the estimated cost of ± 25% did not influence the final result obtained (S3 Table). DAT-LPC is the unique cost-effective test; however, it is not commercially available. To explore a scenario without DAT-LPC, a new analysis was performed considering Kalazar Detect as the baseline or the first comparator, the second least expensive test. Compared to Kalazar Detect, IT LEISH presented ICERs of US$ 1.04 and US$ 13.35 per case correctly diagnosed, using blood or serum samples, respectively. In turn, effectiveness was still improved by the Ridascreen Leishmania Ab test in comparison to IT LEISH at a higher cost, expressed by the ICERs of US$ 52.40 and US$ 36.69 per case correctly diagnosed, using blood and serum samples, respectively (Table 4 –Comparison 2).

**Table 4:** Cost-effectiveness analysis of diagnostic tests for visceral leishmaniasis for HIV infected patients.

**Costs of IT LEISH performed in blood**												
**Diagnostic tests**	**Comparison 1**						**Comparison** 2					
	**C (US$)**	**IC (US$)**	**E**	**IE**	**ICER (US$)**	**DM**	**C (US$)**	**IC (US$)**	**E**	**IE**	**ICER (US$)**	**DM**
DAT-LPC^α^	3.72		0.90				-	-	-	-	-	-
Kalazar Detect	5.01	1.29	0.64	-0.27	*	AbD	5.01		0.64			
IT LEISH	5.12	1.40	0.74	-0.16	*	AbD	5.12	0.11	0.74	0.11	1.04	ND
IFI Leishmaniose Humana	8.06	4.34	0.71	-0.19	*	AbD	8.06	2.94	0.71	-0.04	*	AbD
Ridascreen *Leishmania* Ab	9.53	5.81	0.83	-0.07	*	AbD	9.53	4.41	0.83	0.08	52.40	ND
*Leishmania* ELISA IgG+IgM	11.94	8.22	0.74	-0.16	*	AbD	11.94	2.41	0.74	-0.08	*	AbD
*Leishmania* IFA IgG	12.30	8.58	0.71	-0.19	*	AbD	12.30	2.77	0.71	-0011	*	AbD
NovaLisa *Leishmania infantum* IgG	17.04	13.32	0.76	-0.14	*	AbD	17.04	7.51	0.76	-0.07	*	AbD
	**Cost of all tests performed in serum**											
**Diagnostic tests**	**Comparison 1**						**Comparison 2**					
	**C (US$)**	**IC (US$)**	**E**	**IE**	**ICER (US$)**	**DM**	**C (US$)**	**IC (US$)**	**E**	**IE**	**ICER (US$)**	**DM**
DAT-LPC^α^	3.72		0.90				-	-	-	-	-	-
Kalazar Detect	5.01	1.29	0.64	-0.27	*	AbD	5.01		0.64			
IT LEISH	6.44	2.72	0.74	-0.16	*	AbD	6.44	1.43	0.74	0.11	13.35	ND
IFI Leishmaniose Humana	8.06	4.34	0.71	-0.19	*	AbD	8.06	1.62	0.71	-0.04	*	AbD
Ridascreen *Leishmania* Ab	9.53	5.81	0.83	-0.07	*	AbD	9.53	3.09	0.83	0.08	36.69	ND
*Leishmania* ELISA IgG+IgM	11.94	8.22	0.74	-0.16	*	AbD	11.94	2.41	0.74	-0.08	*	AbD
*Leishmania* IFA IgG	12.30	8.58	0.71	-0.19	*	AbD	12.30	2.77	0.71	-0.11	*	AbD
NovaLisa *Leishmania infantum* IgG	17.04	13.32	0.76	-0.14	*	AbD	17.04	7.51	0.76	-0.07	*	AbD

Comparison 1: including all diagnostic test evaluated for HIV-infected patients; Comparison 2: excluding DAT-LPC from analysis; C: cost (cost value for the diagnostic tests); IC: incremental cost (difference in cost between a diagnostic tests and the previous less costly test on the cost-effectiveness frontier); E: effectiveness (effectiveness value for the diagnostic tests); IE: incremental effectiveness (difference in effectiveness between a diagnostic test sand the previous less costly test on the cost-effectiveness frontier); ICER: incremental cost-effectiveness ratio (the incremental cost-effectiveness ratio comparing a diagnostic tests to the previous less costly test on the cost-effectiveness frontier); DM: dominance; ND: undominated; AbD: absolutely dominated

^α^baseline or first comparator

*negative ICER values.

Cost-effectiveness analyses comparing diagnostic tests according to their methodology are shown in Table 5 and Table 6. Among ELISA tests, Ridascreen Leishmania Ab was the least expensive test and, for HIV-infected patients, it was also the most effective. For HIV-uninfected patients, NovaLisa Leishmania infantum IgG presented an incremental effectiveness, with an ICER of US$ 826.09 per case correctly diagnosed. Among RDTs, IT-LEISH was the most expensive and effective test, presenting different ICERs according to HIV status and biological samples used (blood or serum). Among the immunofluorescence tests, the effectiveness of both tests was the same, while IIF Human Leishmaniasis was the less costly.

**Table 5:** Cost-effectiveness analysis for visceral leishmaniasis diagnostic tests in HIV-uninfected patients, stratified by test methodology.

Diagnostic tests	C (US$)	IC US$)	E	IE	ICE (US$)	DM
I***mmunoenzymatic assays***						
Ridascreen *Leishmania* Ab ^α^	9.53		0.88			
*Leishmania* ELISA IgG+IgM	11.94	2.41	0.83	-0.05	*	AbD
NovaLisa *Leishmania infantum* IgG	17.04	7.51	0.89	0.01	826.09	ND
***Rapid tests***						
OnSite *Leishmania* IgG/IgM Combo (blood)^α^	3.48		0.92			
Kalazar Detect	5.01	1.50	0.94	0.02	111.68	WD
IT LEISH (blood)	5.12	0.11	0.96	0.02	4.76	ND
Kalazar Detect ^α^	5.01		0.94			
OnSite *Leishmania* IgG/IgM Combo (serum)	5.10	0.09	0.92	-0.02	*	AbD
IT LEISH (serum)	6.44	1.43	0.96	0.02	61.18	ND
***Immunofluorescence reactions***						
IIF Human Leishmaniasis ^α^	8.06		0.85			
*Leishmania* IFA IgG	12.30	4.24	0.85	−0.00	*	AbD

**Table 6:** Cost-effectiveness analysis for visceral leishmaniasis diagnostic tests in HIV-infected patients, stratified by test methodology.

Diagnostic tests	C (US$)	IC (US$)	E	IE	ICER (US$)	DM
I***mmunoenzymatic assays***						
Ridascreen *Leishmania* Ab^α^	9.53		0.83			
*Leishmania* ELISA IgG+IgM	11.94	2.41	0.74	-0.08	*	AbD
NovaLisa *Leishmania infantum* IgG	17.04	7.51	0.76	-0.07	*	AbD
***Rapid tests***						
Kalazar Detect^α^	5.01		0.64			
IT LEISH (blood)	5.12	0.11	0.74	0.10	1.04	ND
Kalazar Detect ^α^	5.01		0.64			
IT LEISH (serum)	6.44	1.43	0.74	0.10	13.35	ND
***Immunofluorescence reactions***						
IIF Human Leishmaniasis ^α^	8.06		0.71			
*Leishmania* IFA IgG	12.30	4.24	0.71	0.01	642.97	ND

C: cost (cost value for the diagnostic tests); IC: incremental cost (difference in cost between a diagnostic tests and the previous less costly test on the cost-effectiveness frontier); E: effectiveness (effectiveness value for the strategy); IE: incremental effectiveness (difference in effectiveness between a diagnostic tests and the previous less costly test on the cost-effectiveness frontier); ICER: incremental cost-effectiveness ratio (the incremental cost-effectiveness ratio comparing a diagnostic tests to the previous less costly test on the cost-effectiveness frontier); DM: dominance; ND: undominated; AbD: absolutely dominated; WD: weakly dominant

^α^baseline or first comparator

*negative ICER values.

## Discussion

VL represents a costly problem for the public health system and for society, mainly due to the loss of productivity commonly associated with morbidity and mortality [25]. The reduction of VL impact requires improving the efficiency of the health system as a whole, which ultimately implies correct diagnosis and timely treatment. In this sense, the use of accurate diagnostic tests is especially important for VL, a disease in which the misdiagnosis could be extremely dangerous in both scenarios: a false-positive result would lead to an unnecessary toxic treatment, which in Brazil means antimony derivative in most cases, and a false negative test result would leave untreated patients with a lethal disease [12]. In addition, given the differences in cost and performance of available tests, economic evaluations are useful tools to support decision making, balancing high-quality health care and financing availability.

Our analyses indicate DAT-LPC and IT LEISH as the most cost-effective diagnostic tests. In an ideal scenario, both tests should be available, each one in a specific context. RDTs are simple to perform and to interpret, do not require laboratory infrastructure or specialized professionals and can be performed at the patient’s bedside. Thus, they are ideal tools as point-of-care testing at primary health centers, after an implementation process [26]. However, caution is required for VL screening in subgroups of patients with expected low performance of RDTs: immunosuppressed patients and children under 2 years old [27,28]. Conversely, direct agglutination is a test that fits well into laboratories with a minimum installed infrastructure, to be performed by a trained laboratory professional and indicated for the diagnosis of cases in which the RDTs are insufficient, at a more complex and central level of care [13,29]. According to these findings, methodologies such as RIFI and ELISAs should be deprecated in view of their performance and cost-effectiveness.

HIV infection affects 7.4% of patients with VL in Brazil, which makes a diagnostic strategy directed to this co-infected population of utmost importance [3]. Differently from VL immunocompetent patients who present an exacerbated and specific humoral immune response, HIV co-infected patients exhibit a lower production of specific antibodies due to the absence of T cells, essential for the presentation and stimulation of B cells [30]. Serological tests, in particular the RDTs, have an already recognized low sensitivity for the diagnosis of VL among HIV patients [28]. This observation has a significant impact on the VL investigation algorithms, as it means that more invasive and more expensive approaches need to be employed in the subgroup of HIV-infected patients. With a performance that stands out from other tests, DAT has been shown to be a serological test with the best results among HIV-infected patients [6,28]. According to our results, DAT-LPC was the most cost-effective test in this subgroup of patients. In addition to the cost-effectiveness perspective, in the Brazilian scenario, DAT-LPC may bring national production autonomy, considering that it has been improved in Brazil and is currently in the process of technology transfer to a national pharmaceutical industry, with potential for low-cost production, eliminating import expenses [31,32]. For HIV-uninfected patients, a cost-effectiveness study assessing six different diagnostic tests used in Brazil also showed DAT-LPC as a less costly and a highly accurate (99%) strategy (estimated at US$ 4.92), indicating a cost-effective test [14].

In Brazil, changes of RDTs available in the health public system for VL diagnosis have been common, based only on an evaluation of the test unit cost and not on a cost-effectiveness analysis. Our analysis was conducted in 2018, when OnSite *Leishmania* IgG/IgM Combo was the RDT available in Brazil. Subsequently, in 2019, the LSH Ab ECO (Eco Diagnóstica) replaced OnSite in the Brazilian public health system. However, due to the absence of local accuracy studies at the time of our analysis, LSH Ab ECO (Eco Diagnóstica) was not included in our analysis.

Among the RDTs, IT-LEISH presented low ICER values and should be considered an important cost-effective test independently of HIV status and biological samples used (blood or serum), suggesting that the decision to replace this test with the OnSite *Leishmania* IgG/IgM Combo was not cost-effective. The use of blood as a biological sample represents a great advance in VL diagnosis, especially in remote areas with poor lab infrastructure. In Brazil, despite the availability of RDTs for use with blood/serum and the feasibility of RDT decentralization to municipal laboratories [26], they are still centralized in reference labs to be performed using serum. Therefore, even in referral labs, where several resources are available to carry out other methodologies, RDTs are also used due to their high performance and availability, confirming the usefulness of a global comparison among tests.

In this study, the first cost-effectiveness analysis including all diagnostic tests for VL registered in ANVISA up to 2017 was performed. The strength of the economic analysis carried out involves the measurement of direct costs through the microcosting technique. This approach allows a high degree of detail due to the values for all individual procedures and processes are summed to generate the total direct cost of the evaluated diagnostic test [17]. Another advantage of this study is that the sensitivity and specificity results for the diagnostic tests for patients according to HIV status were obtained from studies conducted in Brazil.

Some limitations need to be observed to interpret these results. DAT-LPC is the only test not commercially available among the compared tests; thus, its total cost can be underestimated, considering that the taxes and logistics freight charges were not computed; however, these additional costs could be compensated by the test’s lower cost of mass production. According to the considered prevalence of the disease, the effectiveness of the tests varies, and consequently, the results of the analysis must be changed. Finally, we have considered the laboratory technician salaries of workers in the city of Belo Horizonte, Minas Gerais, as the reference salary for the test’s costs estimation, which cannot correspond to the realities in other scenarios.

Economic evaluations in health care are of extreme importance, especially in developing countries, where needs always outweigh available resources [33,34]. In Brazil, the Ministry of Health has stimulated the development of economic evaluation studies and applied their results for incorporation of new technologies in the Unified Health System (SUS). The requirements for a technology acquisition are efficacy/accuracy and safety, in addition to the comparative economic assessment of benefits and costs in relation to existing technologies [35]. Despite the usefulness of comparing technologies and studies, the establishment of a unique cost-effectiveness threshold to be applied in the public health system is not universally accepted since it would be unable to capture all the important values for different societies [36,37]. Pichon-Rivière et al. (2017), based on health costs and life expectancy, report that the threshold value for Brazil should be between 0.62–1.05 gross domestic product (GDP) per capita [38]. Considering the value of the Brazilian GDP per capita in 2017 (US$ 7,385.96) [39], the threshold would be in the range of US$ 4,579.29–7,755.26.

The results obtained in this study confirm that cost-effectiveness studies, through a comparative analysis that considers the gain in health in relation to cost, are useful tools to allow the appropriate allocation of resources and in the establishment of an efficient health system. The closer to the reality of application the analyses are conducted, the greater the utility of the conclusions of this type of study. For the Brazilian scenario, one RDT and DAT were the most cost-effective tests for VL diagnosis among non-HIV-infected patients, and the last one was the most cost-effective for HIV-infected patients.

## Supporting information

S1 Table Detailed costs of the items included in the direct cost estimates of the diagnostic test evaluated for visceral leishmaniasis. (DOCX)

S2 Table Sensitivity analyses for diagnostic tests for visceral leishmaniasis for HIV-uninfected patients. (DOCX)

S3 Table Sensitivity analyses for diagnostic tests for visceral leishmaniasis for HIV-infected patients. (DOCX)
