# Supplementary material for: Cost-effectiveness of serological tests for human visceral leishmaniasis in the Brazilian scenario
Source: PLoS Negl Trop Dis. 2020 Oct 8;14(10):e0008741. doi: 10.1371/journal.pntd.0008741 (PMC7544087; doi:10.1371/journal.pntd.0008741)
Supplement: S1 Table — (DOCX) [file pntd.0008741.s001.docx]

**S1 Table. Detailed costs of the items included in the direct cost estimates of the diagnostic test evaluated for visceral leishmaniasis.**

| **Diagnostic test** | **Items included in the cost estimate** | **Cost (US$)** |
| --- | --- | --- |
| **IT LEISH**  **(using blood** **samples)** | Unit value of the test | 3.71 |
|  | Payment for the laboratory technician | 1.28 |
|  | Personal protective equipment | 0.13 |
|  | **Total** | **5.12** |
|  | **Variation ± 25%** | **3.84 – 6.40** |
| **IT LEISH**  **(using serum** **samples)** | Unit value of the test | 3.71 |
|  | Collection of biological material | 0.22 |
|  | Payment for the laboratory technician | 2.18 |
|  | Personal protective equipment | 0.26 |
|  | Consumables | 0.01 |
|  | Equipment maintenance | 0.06 |
|  | **Total** | **6.44** |
|  | **Variation ± 25%** | **4.83 – 8.05** |
| **OnSite *Leishmania* IgG/IgM Combo**  **(using blood** **samples)** | Unit value of the test | 2.37 |
|  | Payment for the laboratory technician | 0.98 |
|  | Personal protective equipment | 0.13 |
|  | **Total** | **3.48** |
|  | **Variation ± 25%** | **2.61 - 4.35** |
| **OnSite *Leishmania* IgG/IgM Combo**  **(using serum** **samples)** | Unit value of the test | 2.37 |
|  | Collection of biological material | 0.22 |
|  | Payment for the laboratory technician | 2.18 |
|  | Personal protective equipment | 0.26 |
|  | Consumables | 0.01 |
|  | Equipment maintenance | 0.06 |
|  | **Total** | **5.10** |
|  | **Variation ± 25%** | **3.82 - 6.37** |
| **IIF Human Leishmaniasis** | Unit value of the test | 0.42 |
|  | Collection of biological material | 0.22 |
|  | Payment for the laboratory technician | 6.83 |
|  | Personal protective equipment | 0.26 |
|  | Consumables | 0.13 |
|  | Equipment maintenance | 0.21 |
|  | **Total** | **8.06** |
|  | **Variation ± 25%** | **6.05 - 10.08** |
| ***Leishmania* IFA IgG** | Unit value of the test | 5.47 |
|  | Collection of biological material | 0.22 |
|  | Payment for the laboratory technician | 6.02 |
|  | Personal protective equipment | 0.26 |
|  | Consumables | 0.13 |
|  | Equipment maintenance | 0.21 |
|  | **Total** | **12.30** |
|  | **Variation ± 25%** | **9.23 - 15.38** |
| ***Leishmania* ELISA IgG+IgM** | Unit value of the test | 4.41 |
|  | Collection of biological material | 0.22 |
|  | Payment for the laboratory technician | 6.66 |
|  | Personal protective equipment | 0.26 |
|  | Consumables | 0.20 |
|  | Equipment maintenance | 0.19 |
|  | **Total** | **11.94** |
|  | **Variation ± 25%** | **8.95 - 14.92** |
| **RIDASCREEN Leishmania Ab** | Unit value of the test | 4.45 |
|  | Collection of biological material | 0.22 |
|  | Payment for the laboratory technician | 4.27 |
|  | Personal protective equipment | 0.26 |
|  | Consumables | 0.20 |
|  | Equipment maintenance | 0.13 |
|  | **Total** | **9.53** |
|  | **Variation ± 25%** | **7.15 - 11.91** |
| **NovaLisa *Leishmania infantum* IgG** | Unit value of the test | 9.18 |
|  | Collection of biological material | 0.22 |
|  | Payment for the laboratory technician | 7.00 |
|  | Personal protective equipment | 0.26 |
|  | Consumables | 0.20 |
|  | Equipment maintenance | 0.19 |
|  | **Total** | **17.04** |
|  | **Variation ± 25%** | **12.78 - 21.31** |
| **Kalazar Detect** | Unit value of the test | 2.49 |
|  | Collection of biological material | 0.22 |
|  | Payment for the laboratory technician | 1.97 |
|  | Personal protective equipment | 0.26 |
|  | Consumables | 0.01 |
|  | Equipment maintenance | 0.06 |
|  | **Total** | **5.01** |
|  | **Variation ± 25%** | **3.76 - 6.26** |
| **DAT - LPC** | Unit value of the test | 0.54 |
|  | Collection of biological material | 0.22 |
|  | Payment for the laboratory technician | 2.13 |
|  | Personal protective equipment | 0.26 |
|  | Consumables | 0.44 |
|  | Equipment maintenance | 0.13 |
|  | **Total** | **3.72** |
|  | **Variation ± 25%** | **2.79 - 4.65** |
