# Supplementary material for: Cost-effectiveness of serological tests for human visceral leishmaniasis in the Brazilian scenario
Source: PLoS Negl Trop Dis. 2020 Oct 8;14(10):e0008741. doi: 10.1371/journal.pntd.0008741 (PMC7544087; doi:10.1371/journal.pntd.0008741)
Supplement: S2 Table — (DOCX) [file pntd.0008741.s002.docx]

**S2 Table.** Sensitivity analyses for diagnostic tests for visceral leishmaniasis for patients not infected with human immunodeficiency virus.

| **Parameter** | **Variable** | | **Non-HIV carriers** |
| --- | --- | --- | --- |
| **All analyzed tests** | | | |
| IT LEISH | Sensitivity ≤ 95% | | IT LEISH ceases to be cost-effective. |
|  | Specificity ≤ 93.9% | | IT LEISH ceases to be cost-effective. |
| OnSite *Leishmania* IgG/IgM Combo | Cost using blood ≥ US$ 3.72 | | OnSite *Leishmania* IgG/IgM Combo ceases to be cost-effective (IT LEISH and OnSite *Leishmania* IgG/IgM Combo in blood). |
|  | Cost using blood ≤ US$ 3.51 | | OnSite *Leishmania* IgG/IgM Combo ceases to be cost-effective (IT LEISH and OnSite *Leishmania* IgG/IgM Combo in blood). |
| DAT-LPC | Sensitivity ≤ 91.5% | | Kalazar Detect becomes cost-effective, with an ICER of US$ 8,569.22. |
|  | Sensitivity ≤ 89.5% | | DAT-LPC ceases to be cost-effective (IT LEISH and OnSite *Leishmania* IgG/IgM Combo in blood). |
|  | Sensitivity ≥ 95% | | IT LEISH ceases to be cost-effective. |
|  | Specificity ≤ 92.9% | | Kalazar Detect becomes cost-effective, with an ICER of US$ 40,168.21. |
| **Immunoenzymatic assays** | | | |
| Ridascreen *Leishmania* Ab | Sensitivity ≤ 89.3% | | *Leishmania* ELISA IgG+IgM becomes cost-effective, with an ICER of US$15,552.73 |
|  | Sensitivity ≥ 98.4% | | NovaLisa *Leishmania infantum* IgG ceases to be cost-effective. |
|  | Specificity ≥ 81.8% | | NovaLisa *Leishmania infantum* IgG ceases to be cost-effective. |
| NovaLisa *Leishmania infantum* IgG | Sensitivity ≤ 86.6% | | NovaLisa *Leishmania infantum* IgG ceases to be cost-effective. |
|  | Specificity ≤ 91.2% | | NovaLisa *Leishmania infantum* IgG ceases to be cost-effective. |
| **Rapid tests** | | | |
| IT LEISH | Sensitivity ≤ 92.5% | | IT LEISH ceases to be cost-effective. |
|  | Cost using blood ≤ US$ 5.00 | | Kalazar Detect ceases to be cost-effective. |
|  | Cost using serum ≤ US$ 5.01 | | Kalazar Detect ceases to be cost-effective. |
| OnSite *Leishmania* IgG/IgM Combo | Cost using serum ≤ US$ 5.01 | | OnSite *Leishmania* IgG/IgM Combo becomes cost-effective, and Kalazar Detect has an ICER of US$ 0.25. |
|  | Sensitivity ≥ 93% | | Kalazar Detect ceases to be cost-effective (IT LEISH and OnSite *Leishmania* IgG/IgM Combo in blood), and OnSite *Leishmania* IgG/IgM Combo becomes cost-effective, with an ICER of US$ 512.52 (IT LEISH and OnSite *Leishmania* IgG/IgM Combo in serum). |
| Kalazar Detect | Cost ≥ US$ 5.12 | | Kalazar Detect ceases to be cost-effective. |
|  | Cost ≥ US$ 5.10 | | OnSite *Leishmania* IgG/IgM Combo becomes cost-effective (US$ 5.10/effectiveness: 92%), and Kalazar Detect presents an ICER of US$ 0.14. |
|  | Sensitivity ≤ 91% | | Kalazar Detect ceases to be cost-effective (IT LEISH and OnSite *Leishmania* IgG/IgM Combo in blood), and OnSite *Leishmania* IgG/IgM Combo becomes cost-effective, with an ICER of US$ 427.10 (IT LEISH and OnSite *Leishmania* IgG/IgM Combo in serum). |
|  | Sensitivity ≥ 96.5% | | IT LEISH ceases to be cost-effective. |
|  | Specificity ≤ 90.9% | | Kalazar Detect ceases to be cost-effective (IT LEISH and OnSite *Leishmania* IgG/IgM Combo in blood), and OnSite *Leishmania* IgG/IgM Combo becomes cost-effective, with an ICER of US$ 1,807.30 (IT LEISH and OnSite *Leishmania* IgG/IgM Combo in serum). |
| **Indirect immunofluorescence reactions** | | | |
| IIF Human Leishmaniasis | | Sensitivity ≤ 85.9% | *Leishmania* IFA IgG becomes cost-effective, with an ICER of US$ 78,951.06. |
|  |  | Specificity ≤ 81.8% | *Leishmania* IFA IgG becomes cost-effective, with an ICER of US$ 99,849.87. |
| *Leishmania* IFA IgG | | Sensitivity ≥ 79.1% | *Leishmania* IFA IgG becomes cost-effective, with an ICER of US$ 60,193.18. |
|  |  | Specificity ≥ 96.2% | *Leishmania* IFA IgG becomes cost-effective, with an ICER of US$ 99,849.87. |

**Legend:** incremental cost-effectiveness ratio (ICER).
