# Supplementary material for: Cost-effectiveness of serological tests for human visceral leishmaniasis in the Brazilian scenario
Source: PLoS Negl Trop Dis. 2020 Oct 8;14(10):e0008741. doi: 10.1371/journal.pntd.0008741 (PMC7544087; doi:10.1371/journal.pntd.0008741)
Supplement: S3 Table — (DOCX) [file pntd.0008741.s003.docx]

**S3 Table.** Sensitivity analyses for diagnostic tests for visceral leishmaniasis for patients infected with human immunodeficiency virus.

| **Parameter** | **Variable** | **HIV carriers** |
| --- | --- | --- |
| **All analyzed tests** | | |
| DAT-LPC | Sensitivity ≤ 79% | Ridascreen *Leishmania* Ab presents an ICER of US$ 17321.74. |
| **Immunoenzymatic assays** | | |
| Ridascreen *Leishmania* Ab | Sensitivity ≤ 71.4% | NovaLisa *Leishmania* *infantum* IgG presents an ICER of US$ 44547.56. |
|  | Sensitivity ≤ 68.4% | *Leishmania* ELISA IgG+IgM presents an ICER of US$ 35064.33. |
| NovaLisa *Leishmania* *infantum* IgG | Sensitivity ≥ 76.6% | NovaLisa *Leishmania* *infantum* IgG becomes cost-effective, with an ICER of US$ 44547.56. |
| **Rapid tests** | | |
| IT LEISH | Sensitivity ≤ 47% | IT LEISH ceases to be cost-effective. |
|  | Cost using blood ≤ US$ 5.00 | Kalazar Detect ceases to be cost-effective. |
|  | Cost using serum ≤ US$ 5.01 | Kalazar Detect ceases to be cost-effective. |
| Kalazar Detect | Sensitivity ≥ 63% | IT LEISH ceases to be cost-effective. |
|  | Cost ≥ US$ 5.12 | Kalazar Detect ceases to be cost-effective (IT LEISH in blood). |
| **Indirect immunofluorescence reactions** | | |
| IIF Human Leishmaniasis | Sensitivity ≥ 62% | *Leishmania* IFA IgG ceases to be cost-effective. |
|  | Specificity ≥ 92% | *Leishmania* IFA IgG ceases to be cost-effective. |
| *Leishmania* IFA IgG | Sensitivity ≤ 60% | *Leishmania* IFA IgG ceases to be cost-effective. |
|  | Specificity ≤ 90% | *Leishmania* IFA IgG ceases to be cost-effective. |

**Legend:** incremental cost-effectiveness ratio (ICER).
